# Anesthetic Management During Total Abdominal Hysterectomy in a Patient With Cold Agglutinin Disease: A Case Report

**DOI:** 10.7759/cureus.83217

**Published:** 2025-04-29

**Authors:** Erika Imasato, Kakeru Okubo, Junko Tamura, Yuko Nishiwaki, Hideya Kato

**Affiliations:** 1 Department of Anesthesiology, Saiseikai Shiga Hospital, Shiga, JPN

**Keywords:** amino acid infusion, autoimmune hemolytic anemia, cold agglutinin disease, temperature management, thermal amplitude

## Abstract

Cold agglutinin disease (CAD) is a very rare autoimmune disorder characterized by hemolytic anemia triggered by cold exposure or hypothermia. Thrombotic complications may ensue, with potentially fatal outcomes. The lowering of body temperature under general anesthesia poses risks of triggering agglutination and hemolysis. A 42-year-old female patient with CAD was scheduled to undergo a full abdominal hysterectomy for uterine fibroids. Strict temperature management was implemented in anticipation of hypothermia because extensive abdominal exposure to room air would be required during open surgery. Her core and surface temperatures were monitored via rectal and axillary temperature measurements. To keep her body temperature stably elevated, an amino acid infusion was administered preoperatively, the operating room temperature was set to 30°C, and fluid and forced-air warming devices were used intraoperatively. Throughout the surgery, both core and surface temperatures were successfully maintained at 37°C -38°C, and anesthesia was concluded without the occurrence of hemolysis.

## Introduction

Cold agglutinin disease (CAD) is a type of autoimmune hemolytic anemia (AIHA) in which autoantibodies that react with erythrocyte membrane antigens are produced in response to cold stimuli [1,2]. This immune reaction causes erythrocytes to agglutinate and hemolyze, leading to anemia and peripheral circulatory damage, which may subsequently precipitate fatal thrombotic complications [1,2].

Under general anesthesia, central body heat migrates toward peripheral tissues, with consequent vasodilation of arterioles and other resistance vessels near the body surface. This redistribution of heat and significant heat loss from the skin surface result in rapid central cooling [3,4]. This phenomenon puts patients with CAD under general anesthesia at great risk of falling below the thermal amplitude (i.e., the highest temperature at which agglutination occurs), exposing them to temperatures at which autoantibodies are activated, and setting off the cascade of erythrocyte agglutination and hemolysis. Therefore, maintaining warmth by preventing heat loss from the body surface is crucial to preventing hemolytic episodes in patients with CAD under general anesthesia.

We present the case of a female patient with CAD who required general anesthesia for a full abdominal hysterectomy, which was completed successfully without inducing hemolysis by implementing comprehensive measures to control body temperature.

## Case presentation

A 42-year-old female patient presented with lower abdominal pain and irregular genital bleeding from several days prior to admission. Her condition worsened, including dizziness, respiratory distress, and difficulty moving, whereupon she received emergency transport to our hospital. A detailed examination revealed large uterine fibroids, and a full abdominal hysterectomy was scheduled. Initial blood tests indicated a hemoglobin (Hb) level of 5.6 g/dL (11.6-14.8 g/dL), hematocrit of 11.6% (35.1%-44.4%), total bilirubin level of 3.51 mg/dL (0.4-1.5 mg/dL), and direct bilirubin level of 0.35 mg/dL (0.00-0.20 mg/dL), suggesting hemolytic anemia. Based on the results of further tests-a positive direct Coombs test yielding a cold agglutinin titer of 1:256 and a positive direct antiglobulin test-she was diagnosed with CAD; beforehand, no symptoms specific to CAD, such as thrombosis, had been observed.

Blood tests also revealed anemia, which was thought to be caused by a large uterine fibroid; however, hemolytic anemia associated with cold agglutination could not be ruled out. In addition, in preparation for a full abdominal hysterectomy under general anesthesia, strict temperature management was planned owing to concerns about expected heat loss from the peripheral redistribution of heat as well as decreased core body temperature from opening the abdomen.

Anesthesia course

On the day of admission, four units of concentrated red blood cells were transfused to restore the patient’s Hb level. On the day of surgery (third day of hospitalization), before she entered the operating room (OR), a comprehensive amino acid preparation (Amiparen^®^, containing 20 g of total free amino acids; Otsuka Pharmaceutical, Tokyo, Japan) was infused over a period of two hours to help maintain the perioperative body temperature. The patient's temperature on the ward before entering the OR was 36.7°C.

General anesthesia was administered in addition to thoracic epidural anesthesia. An epidural catheter was inserted between the 12th thoracic and first lumbar vertebrae. Anesthesia induction and tracheal intubation were performed using propofol, remifentanil, and rocuronium. Anesthesia was maintained with continuous administration of 4%-5% desflurane and 0.05 μg/kg/min remifentanil, along with levobupivacaine, through an epidural catheter. Body temperature was monitored via both core (rectal) and surface (axillary) temperatures.

Body temperature was maintained by setting the OR temperature to the possible maximum, i.e., 30°C, and warming the patient’s upper body using a forced-air warming device (Level 1 Convective Warmer^®^; Smiths Medical Japan, Tokyo, Japan). In addition, all infusions were warmed using a fluid warming device (Level 1 Hotline^®^; Smiths Medical Japan).

**Figure 1:**
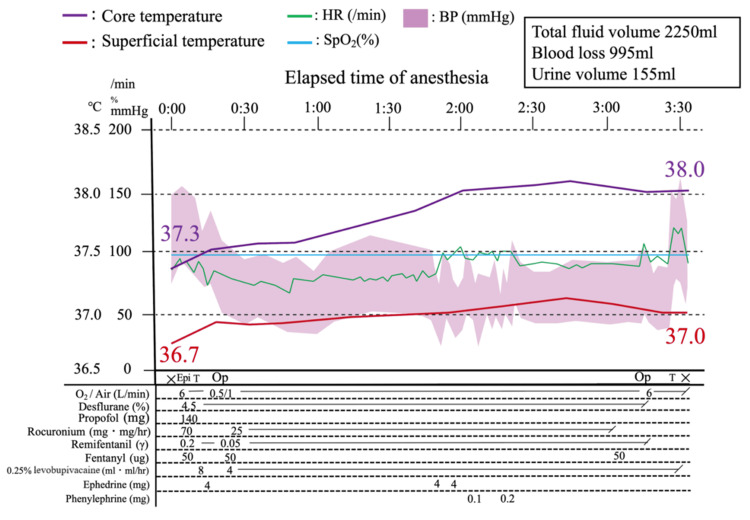
Anesthesia record ×: start and end of anesthesia; Op: start and end of the operation; Epi: epidural anesthesia; T: tracheal intubation and extubation; HR: heart rate; SpO_2_: peripheral capillary oxygen saturation; BP: blood pressure

The intraoperative temperature changes and the anesthesia timeline are shown in Figure 1. After anesthesia induction, both rectal and axillary temperatures showed an upward trend and were 38.0°C and 37.0°C at the time of OR departure, respectively. During surgery, no signs of hemoglobinuria or peripheral circulatory failure, such as cyanosis of the extremities, were noted, and neither of the monitored temperature values dropped below the thermal amplitude. The surgery lasted two hours and 47 minutes, while anesthesia was maintained for three hours and 33 minutes. The total fluid volume infused was 2,250 mL, with blood loss of 995 mL and urine output of 155 mL. The following day, blood tests showed a decrease in Hb levels (6.9 g/dL (11.6-14.8 g/dL)), which was attributed to intraoperative bleeding and hemodilution due to extracellular fluid replacement. The patient’s Hb level was subsequently restored under observation with oral iron supplementation. No signs of hemolysis progression were observed after the operation, and the patient was discharged on postoperative day 8 without any complications.

## Discussion

AIHA can be classified into two types: anemias mediated by warm autoantibodies and those mediated by cold autoantibodies [1,2]. CAD belongs to the latter group. CAD is an extremely rare disease, with an estimated incidence of 0.5-1.9 cases per million individuals, with slight variation by country and region [5].

The mechanism of CAD involves the binding of immunoglobulin M cold autoantibodies to the I antigen on the surface of erythrocytes. The resulting agglutination then activates the complement system, leading to extravascular hemolysis as cells are phagocytosed and destroyed by reticuloendothelial cells bearing C3b receptors [1,2]. Clinically, erythrocyte agglutination results in anemia and peripheral circulatory disturbances, which can lead to fatal thrombotic complications due to microvascular occlusion.

In severe cases, cold exposure from air conditioning can cause cyanosis of the extremities, the tip of the nose, and the ears, even in summer, resulting in hemolysis, hemoglobinuria, and anemia progression [1,2]. Treatment options for CAD include steroid administration, targeted molecular therapies, and plasma exchange [1,2,6]. The temperature range in which cold agglutinins are active is a crucial factor in hemolysis. Therefore, maintaining a body temperature above the thermal amplitude is fundamental in the management of CAD [1,2,7].

The classical decrease in body temperature during general anesthesia can be divided into three phases. In Phase 1, anesthesia lowers the threshold temperature for the cold response in the hypothalamus, significantly inhibiting the vasoconstriction response. This leads to peripheral vasodilation, which marks the start of heat dissipation as central heat is transferred to peripheral tissues via the bloodstream. This redistributive hypothermia causes a rapid decrease in core temperature between 30 and 60 minutes after anesthesia induction. During Phase 2, as the body’s thermoregulatory mechanisms are suppressed by anesthesia, a vasoconstrictive response is prevented despite the reduced core temperature, which thus continues to fall as body heat is lost from peripheral tissues through the skin. In Phase 3, once the core temperature drops below the cold response threshold, the body’s thermoregulatory mechanisms are reactivated, inducing a vasoconstrictive response that reduces heat dissipation from the skin. This stabilizes the thermal equilibrium and brings the core temperature to a near plateau [3,4].

Given the distinct mechanisms by which the body temperature decreases in each of the three phases, comprehensive strategies-including measures to promote heat production, maintain core temperature, and prevent redistributive hypothermia-are necessary for perioperative thermal management of patients with CAD [8,9]. Table 1 shows a list of preoperative and intraoperative measures recommended for this clinical population.

**Table 1:** Perioperative management of patients with cold agglutinin disease CT: computed tomography; MRI: magnetic resonance imaging

Preoperative management	
Infusion	Recommend amino acid and fructose infusions
Examination	The following tests are recommended for confirming thrombotic complications: lower extremity venous echo, chest contrast-enhanced CT scan, head MRI scan, blood test (D-dimer level)
Management in the operating room	
Preparation drug	Prepare haptoglobin for possible hemolytic attacks
Body temperature monitoring	Monitor core temperature as well as palmar temperature
Operating room temperature	Set as high as possible
Infusions	Integrate a warming device into the infusion circuit
Drugs	Avoid using acetaminophen preparations if possible, as they have a body temperature-lowering effect

In patients with CAD, even if the core temperature remains stable, a drop in the peripheral temperature can trigger hemolysis due to the cold response (peripheral vasoconstriction) at the skin surface. In the present case, we monitored both core and peripheral temperatures to ensure that both temperatures were stably maintained, without any dangerous reduction in either.

Amino acid infusions prevent decreases in body temperature during anesthesia [10]. Their administration approximately 1-2 hours before anesthesia induction is considered most effective [11,12]. One known mechanism for the warming effect of amino acid infusions is the direct action of amino acids on the temperature control center of the hypothalamus, which elevates the threshold temperature for the thermoregulatory response, resulting in an increase in core temperature [13]. Other reported mechanisms include increased blood insulin levels due to amino acid infusion, which enhances skeletal muscle protein synthesis and thereby increases heat production [14]. In the present case, the presence of an upward trend-rather than a decline-in both core and surface temperatures may suggest that metabolic heat production exceeded heat loss, an effect we believe was likely attributable to the thermoregulatory benefits of the amino acid infusion.

In addition, among carbohydrates, fructose provides the highest diet-induced thermogenesis. Similar to amino acids, preoperative administration of fructose elevates energy metabolism and raises the vasoconstriction threshold, preventing the body temperature from dropping during anesthesia [15]. Although not used in this case, we believe that fructose infusion may be beneficial in similar cases.

Since epidural analgesia carries a risk of exacerbating heat loss, peripheral nerve block was considered an alternative [16]. However, as the case involved open abdominal surgery, we opted for epidural analgesia for two reasons: earlier mobilization is achieved with high-quality epidural anesthesia than with peripheral nerve block, potentially reducing the length of the patient’s hospital stay, and the comprehensive temperature management strategies employed were expected to satisfactorily prevent hypothermia [17].

Patients with CAD have an elevated risk of venous thromboembolism due to intravascular agglutination [18]. Preoperative screening for deep vein thrombosis using lower limb venous ultrasound-although not performed in the present case-should be considered to prevent fatal complications, such as pulmonary embolism. Moreover, previous reports have noted that thrombi can obstruct peripheral venous lines, suggesting that it may be prudent to use more venous lines than usual or set up a central venous line in such cases [18]. In the current case, we secured two peripheral venous lines and one arterial line from the radial artery for the management of anesthesia. Evaluating and maintaining the patient’s thermal amplitude ahead of surgery is paramount to perioperative management and outcomes, which may affect their daily lives. Meanwhile, even if the surface temperature is maintained at values above the thermal amplitude observed during general anesthesia, the temperature of the extremities may be lower, creating a risk of a cold aggregation reaction in the peripheral blood vessels. Therefore, keeping the patient as warm as possible, within suitable limits, is key. Given the mechanism of hypothermia during general anesthesia described above, the patient's temperature before entering the OR or that after the induction of anesthesia should be used as a threshold for interventions, which should be initiated when the temperature falls below this threshold. In the present case, while the surface temperature was monitored at the armpits, upon reflection, we consider that it should have been monitored at the palms as well. In the literature, intraoperative temperature management involved non-invasive approaches such as warming of infusions, increasing the temperature of the OR, providing amino acid infusions, and using warming blankets [17,18]. Herein, we also chose to use non-invasive approaches to keep the patient warm.

In addition, cases of heart surgery requiring cardiopulmonary bypass have been reported, whereby the procedure was performed at a normal temperature, successfully avoiding perioperative cold aggregation reactions [19].

A drop in body temperature during anesthesia that leads to hemolysis raises the risk of renal tubular necrosis because of free Hb. Practitioners should be vigilant for signs of the onset of hemoglobinuria: if it occurs, they should endeavor to protect the kidneys by administering haptoglobin preparations and increasing renal preload by means of extracellular fluid loading [20].

## Conclusions

Cold aggregation reactions can sometimes lead to fatal outcomes. Preoperative evaluation and perioperative temperature management plans are paramount. Our case of a patient with CAD demonstrated that general anesthesia for total abdominal hysterectomy could be successfully performed without perioperative complications by implementing strict and comprehensive measures to monitor and control body temperature.
